# Dr. John Lizars

**Published:** 1860-07

**Authors:** 


DR. JOHN LIZARS.—It is with deep regret that we are called upon to chronicle the decease of this distinguished gentleman, one of the first surgeons that Scotland has ever produced. On Sunday, the twentieth of May, Dr. Lizars lay down, complaining of being wearied, but symptoms of compression of the brain soon ensued, and he died early the following evening. In our next issue we will present our readers with a brief sketch of his long and well-spent life.

